# Supplementary material for: Scattered slice SHARD reconstruction for motion correction in multi-shell diffusion MRI
Source: Neuroimage. 2021 Jan 15;225:117437. doi: 10.1016/j.neuroimage.2020.117437 (PMC7779423; doi:10.1016/j.neuroimage.2020.117437)
Supplement: Supplementary Data S1 — Supplementary Raw Research Data. This is open data under the CC BY license http://creativecommons.org/licenses/by/4.0/ [file mmc1.pdf]

Scattered slice SHARD reconstruction for motion correction in multi-shell diffusion MRI: Supplementary figures

Daan Christiaens[a,b,c,*], Lucilio Cordero-Grande[a,b,d], Maximilian Pietsch[a,b], Jana Hutter[a,b], Anthony N. Price[a,b], Emer J. Hughes[a], Katy Vecchiato[a], Maria Deprez[a,b], A. David Edwards[a,e], Joseph V. Hajnal[a,b], J-Donald Tournier[a,b]

[a] *Centre for the Developing Brain, School of Biomedical Engineering & Imaging Sciences, King's College London, London, U.K.*
[b] *Biomedical Engineering Department, School of Biomedical Engineering & Imaging Sciences, King's College London, London, U.K.*
[c] *Department of Electrical Engineering, ESAT/PSI, KU Leuven, Leuven, Belgium*
[d] *Biomedical Image Technologies, ETSI Telecomunicacin, Universidad Politcnica de Madrid & CIBER-BBN, Madrid, Spain*
[e] *MRC Centre for Neurodevelopmental Disorders, King's College London, London, U.K.*

---

[*] Corresponding author
Email address: daan.christiaens@kcl.ac.uk
(Daan Christiaens)

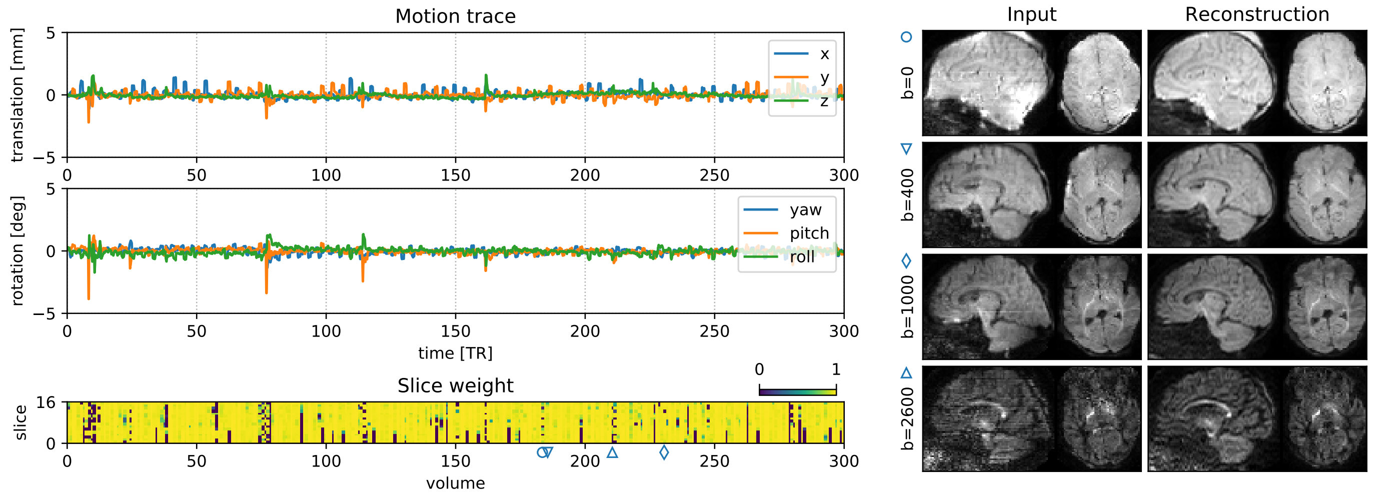

Figure S.1: Motion correction output in Scan 1 (5th percentile). The graphs on the left plot the subject motion (translation and rotation in nautical angles) over time (top), and the weight of each slice in the fit (bottom). Bursts of sudden motion coincide with increased prevalence of outlier slices. The images on the right show example volumes before and after correction. One volume per shell is shown, selected at the 10-percentile of the total slice weight per shell. The icons (○, ▽, ◊, △) indicate the position of the acquired volume in the dMRI series.

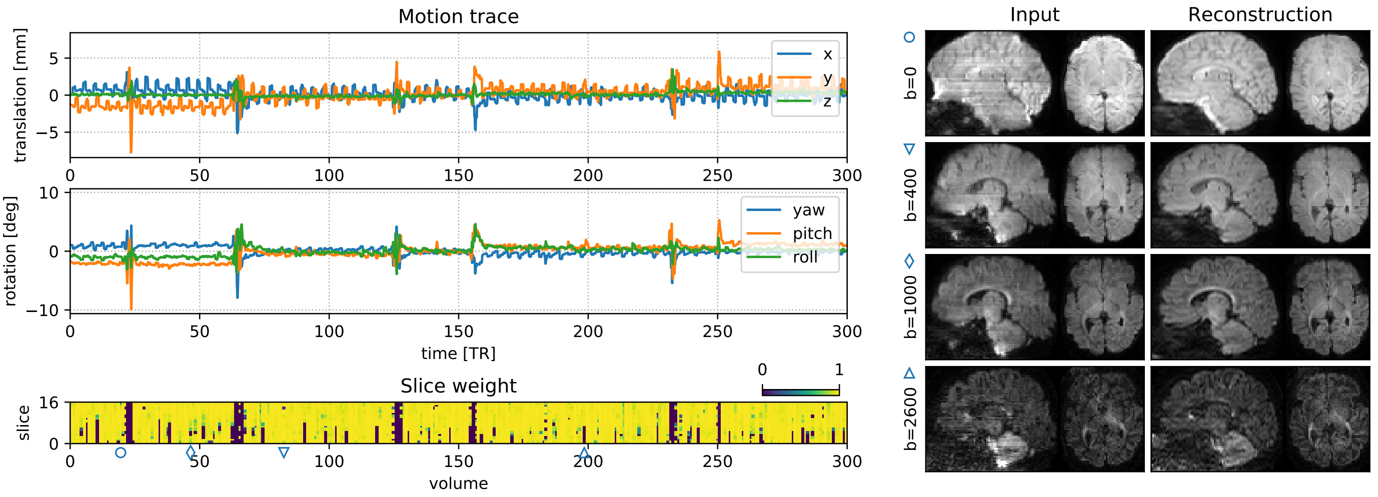

Figure S.2: Motion correction output in Scan 3 (50th percentile). The graphs on the left plot the subject motion (translation and rotation in nautical angles) over time (top), and the weight of each slice in the fit (bottom). Bursts of sudden motion coincide with increased prevalence of outlier slices. The images on the right show example volumes before and after correction. One volume per shell is shown, selected at the 10-percentile of the total slice weight per shell. The icons (○, ▽, ◊, △) indicate the position of the acquired volume in the dMRI series.

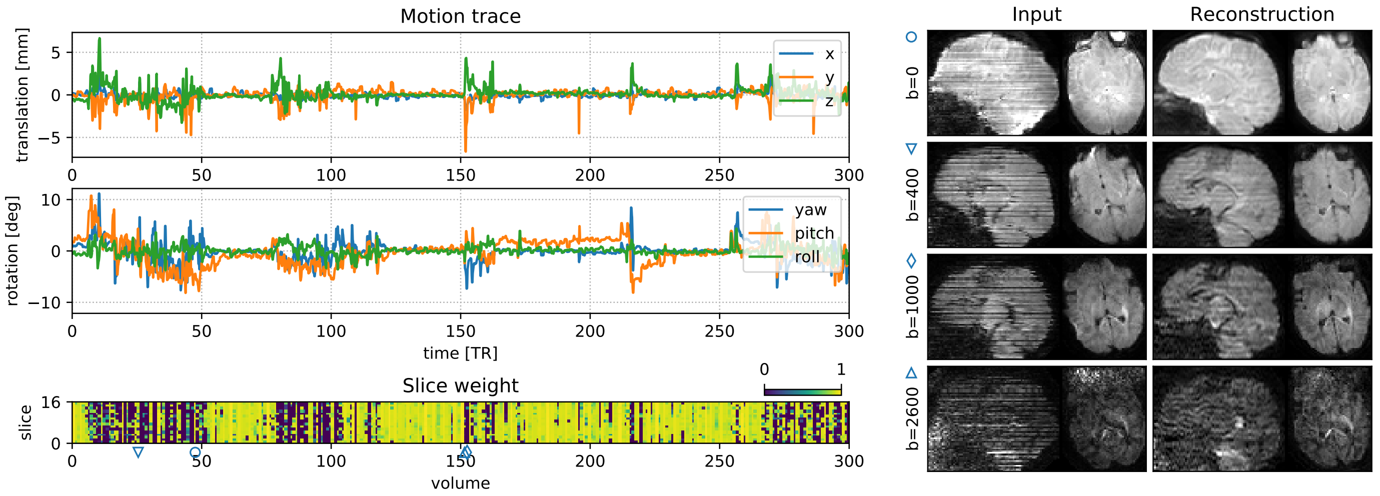

Figure S.3: Motion correction output in Scan 4 (75th percentile). The graphs on the left plot the subject motion (translation and rotation in nautical angles) over time (top), and the weight of each slice in the fit (bottom). Bursts of sudden motion coincide with increased prevalence of outlier slices. The images on the right show example volumes before and after correction. One volume per shell is shown, selected at the 10-percentile of the total slice weight per shell. The icons (○, ▽, ♢, △) indicate the position of the acquired volume in the dMRI series.

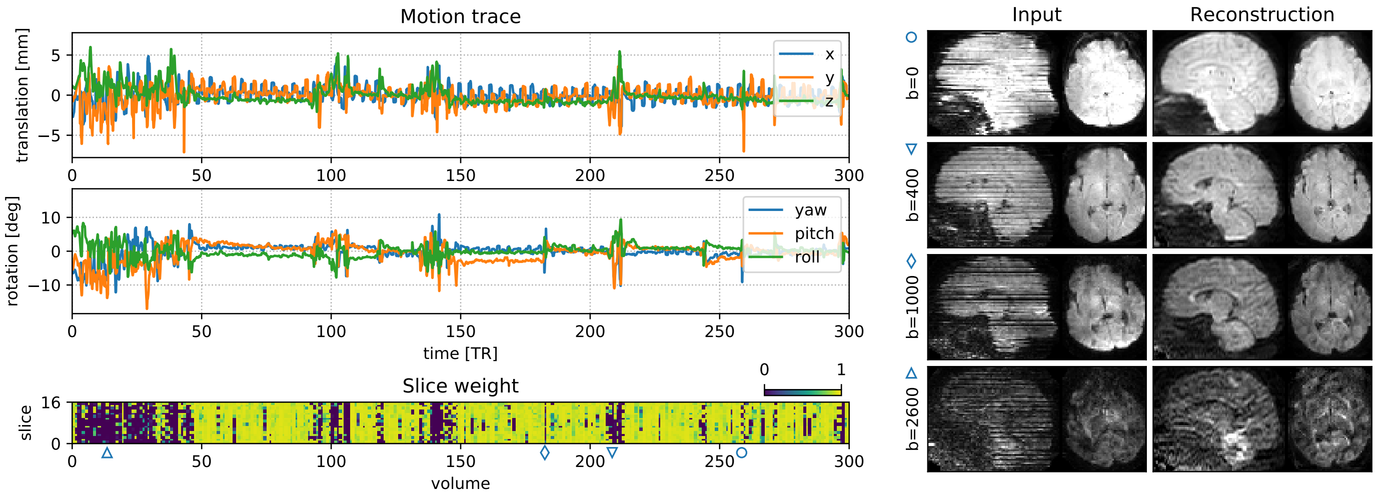

Figure S.4: Motion correction output in Scan 5 (95th percentile). The graphs on the left plot the subject motion (translation and rotation in nautical angles) over time (top), and the weight of each slice in the fit (bottom). Bursts of sudden motion coincide with increased prevalence of outlier slices. The images on the right show example volumes before and after correction. One volume per shell is shown, selected at the 10-percentile of the total slice weight per shell. The icons (○, ▽, ♢, △) indicate the position of the acquired volume in the dMRI series.
